# Canine-Inspired Chemometric Analysis of Volatile Organic Compounds in Urine Headspace to Distinguish Prostate Cancer in Mice and Men

**DOI:** 10.3390/cancers15041352

**Published:** 2023-02-20

**Authors:** Mark Woollam, Amanda P. Siegel, Adam Munshi, Shengzhi Liu, Sunil Tholpady, Thomas Gardner, Bai-Yan Li, Hiroki Yokota, Mangilal Agarwal

**Affiliations:** 1 Integrated Nanosystems Development Institute, Indiana University-Purdue University Indianapolis, Indianapolis, IN 46202, USA; 2 Department of Chemistry and Chemical Biology, Indiana University-Purdue University Indianapolis, Indianapolis, IN 46202, USA; 3 Department of Pharmacology, School of Pharmacy, Harbin Medical University, Harbin 150081, China; 4 Richard L Roudebush Veterans Affairs Medical Center, Indianapolis, IN 46202, USA; 5 Department of Urology, Indiana University School of Medicine, Indianapolis, IN 46202, USA; 6 Department of Biomedical Engineering, Indiana University-Purdue University Indianapolis, Indianapolis, IN 46202, USA; 7 Department of Mechanical and Energy Engineering, Indiana University-Purdue University Indianapolis, Indianapolis, IN 46202, USA

**Keywords:** prostate cancer biomarkers, volatile organic compounds (VOCs), gas chromatography (GC), mass spectrometry (MS), solid phase microextraction (SPME), chemometric analysis

## Abstract

**Simple Summary:**

Volatile organic compounds (VOCs) in urine headspace have been previously shown to be potential biomarkers for prostate cancer. The aim of the current study is to further evaluate urinary VOCs as biomarkers in humans, assess their ability to stratify aggressive tumors, and compare them to the results of murine models of induced prostate cancer. Chemometric analyses were implemented and showed that VOCs in mouse urine were highly dysregulated by prostate cancer and could perfectly distinguish tumor-bearing mice. VOCs in human urine could not only classify any type of prostate cancer with moderate accuracy but could separate aggressive grades with higher sensitivity and specificity. Lastly, there was an overlap in VOC structure and functionality between the mouse and human urine analyses which shows the merit of utilizing murine models for identifying candidate VOC biomarkers for cancer.

**Abstract:**

Canines can identify prostate cancer with high accuracy by smelling volatile organic compounds (VOCs) in urine. Previous studies have identified VOC biomarkers for prostate cancer utilizing solid phase microextraction (SPME) gas chromatography–mass spectrometry (GC-MS) but have not assessed the ability of VOCs to distinguish aggressive cancers. Additionally, previous investigations have utilized murine models to identify biomarkers but have not determined if the results are translatable to humans. To address these challenges, urine was collected from mice with prostate cancer and men undergoing prostate cancer biopsy and VOCs were analyzed by SPME GC-MS. Prior to analysis, SPME fibers/arrows were compared, and the fibers had enhanced sensitivity toward VOCs with a low molecular weight. The analysis of mouse urine demonstrated that VOCs could distinguish tumor-bearing mice with 100% accuracy. Linear discriminant analysis of six VOCs in human urine distinguished prostate cancer with sensitivity = 75% and specificity = 69%. Another panel of seven VOCs could classify aggressive cancer with sensitivity = 78% and specificity = 85%. These results show that VOCs have moderate accuracy in detecting prostate cancer and a superior ability to stratify aggressive tumors. Furthermore, the overlap in the structure of VOCs identified in humans and mice shows the merit of murine models for identifying biomarker candidates.

## 1. Introduction

Current screening methods for prostate cancer include digital rectal exams (DREs), assessing risk factors including family history, and measuring prostate-specific antigen (PSA) levels in blood samples [1]. However, these screening methods have limited sensitivity/specificity and cannot determine tumor aggressiveness [2,3]. With these deficiencies, the results from PSA tests often lead to overtreatment and unnecessary biopsies [4]. Confirmatory diagnostics through biopsy are utilized after prostate cancer screening for pathological grading. Many patients are diagnosed with low grades of prostate cancer and undergo active surveillance where they are routinely biopsied every 6–12 months. Therefore, it is of significant clinical interest to develop a noninvasive and accurate screening method to detect patients with prostate cancer and differentiate indolent from aggressive tumors. Researchers are seeking alternative methods to screen for prostate cancer through identifying biomarkers using various “omic” techniques. These include genomics [5], transcriptomics [6,7], proteomics [8,9], metabolomics [10,11,12], and lipidomics [13,14,15]. One emerging urine-based assay is called SelectMDx, which monitors the expression of two strands of RNA to identify men who would benefit from a biopsy [16]. The benefits of this assay include high diagnostic specificity for patients with advanced grades of prostate cancer (Gleason score (GS) ≥ 7 or International Society of Urological Pathology (ISUP) grade ≥ 3). However, additional biomarkers could complement and improve the ability of prostate cancer screening.

Among some of the most promising and important lines of research include implementing liquid biopsy, which has the ability to measure circulating tumor cells (CTCs), cell-free DNA, exosomal miRNA, and circulating DNA. These methods have been extensively studied for prostate cancer prognosis and diagnosis [17,18,19]. One example of this is androgen-receptor splice variant 7 (AR-V7), phosphatase and tensin homolog (PTEN), and total CTC counts have been previously shown to be significantly altered by advanced prostate cancer and therefore may be able to provide useful prognostic information [20]. Another example is several strands of exosomal miRNA (miR-141-3p, miR-375, miR-21, miR-141, and miR-375) have been correlated with prostate cancer metastasis to the bone [21,22,23]. Previous studies have also shown the outstanding capability of canines to detect prostate cancer by smelling volatile organic compounds (VOCs) in urine headspace. The results have demonstrated that canines can detect prostate cancer more accurately than any other current biological assay. For example, Taverna et al. showed that canines could identify prostate cancer with over 97% sensitivity and specificity [24].

The results from canines have inspired researchers to identify VOC biomarkers emanating from urine samples. Headspace analysis coupled to gas chromatography-mass spectrometry (GC-MS) is the gold standard for VOC biomarker identification. In 2015, Khalid et al. identified a panel of four VOCs in urine headspace using solid phase microextraction (SPME) and GC-MS that could identify prostate cancer with 65% accuracy [25]. More recently in 2019, Lima et al. utilized headspace SPME GC-MS to identify VOC biomarkers for prostate cancer. This study pretreated urine with NaCl and utilized divinylbenzene/carboxen/polydimethylsiloxane (DVB/CAR/PDMS) SPME fibers. This method detected six VOCs that had higher accuracy than previous analyses (sensitivity = 89% and specificity = 83%) [26]. A follow-up study identified that some of the same VOCs, along with other analytes, could distinguish prostate cancer from bladder and kidney cancer with 92% accuracy [27]. The sensitivity and specificity values of different VOC studies for prostate cancer are compared to other previously published methods that identify other non-volatile molecular types of biomarkers (lipids, metabolites, proteins, etc.) [28,29,30,31,32,33,34,35,36,37,38,39,40,41,42,43,44,45,46] in Supplementary Table S1. Non-volatile analytes are present in various biological sample types and have been widely studied as potential biomarkers for prostate cancer. These non-volatiles can be utilized for prostate cancer diagnostics/prognostics and can be more easily tied to biological processes or pathways relative to VOCs alone. However, these non-volatile analytes are not the molecular components that canines detect or smell for accurate prostate cancer stratification.

Taken as a whole, canine ability to detect prostate cancer along with previously published studies implementing GC-MS analysis demonstrate the potential of VOCs to be clinically useful biomarkers for prostate cancer. Even though there are many studies that identify VOCs that can classify any type of prostate cancer, to the knowledge of the authors, there are no investigations dedicated to analyzing VOCs for identifying aggressive prostate cancer. Additionally, previous studies have utilized murine models to identify VOC biomarkers of cancer [47,48,49] but have not demonstrated if the results are translatable to humans. Herein, urine specimens were collected from patients prior to undergoing prostate cancer biopsy or radical prostatectomy. In parallel, urine was also collected from mice with induced prostate cancer. The samples were analyzed through headspace analysis SPME coupled to GC-MS to identify VOCs in urine samples. Chemometric analyses were implemented to identify VOCs differentially expressed due to any form of prostate cancer and between indolent and aggressive prostate cancer samples.

## 2. Materials and Methods

### 2.1. Materials and Instrumentation

UTAK drug-free normal urine, Parafilm, pH paper, and polypropylene cups for urine collection were purchased from ThermoFisher Scientific (Waltham, MA, USA). Sodium hydroxide (50% wt. in solution, nitrogen flushed extra pure) and sodium chloride (99.85% pure) were also purchased from ThermoFisher Scientific. Guanidine hydrochloride (GHCl; pH = 8.5) was purchased from Sigma Aldrich (St. Louis, MO, USA). SPME fibers coated with DVB/CAR/PDMS (two centimeters in length) were purchased from Supelco (Bellefonte, PA, USA). SPME arrows of a similar chemical composition (DVB/Carbon Wide Range/PDMS) were obtained from Restek (Bellefonte, PA, USA). Headspace vials (10 mL) with screw-on caps were purchased from Restek or Agilent (Santa Clara, CA, USA). An Agilent 7890A GC system coupled to an Agilent 7200 MS quadrupole time-of-flight (QTOF) equipped with a PAL autosampling system (CTC Analytics, Zwingen, Switzerland) was used to incubate, extract, and analyze the VOCs. The GC column utilized for VOC separation was a Restek Rxi-5ms column of 30 m in length, a 0.25 mm internal diameter, and a 0.25 μm film thickness.

### 2.2. Patient Recruitment

The inclusion criteria for sample collection from human subjects included the following: veterans at the Richard L. Roudebush Veteran’s Affairs Medical Center (VAMC) in Indianapolis recommended for prostate cancer biopsy, aged 40–85 years old, and a small set of men who were scheduled for radical prostatectomy. The exclusion criteria included serious additional medical problems (including another form of cancer within the last five years) or previous treatment for prostate cancer. The patients provided consent and urine samples were collected on the same day. Institutional Review Board (1506963798) and R&D permission from Indiana University and the VAMC were obtained. The samples were deidentified and classified by ISUP grades ranging from 0 to 5, including samples scored 0 (no cancer identified), 1 (the cancer cells found were predominantly indolent), 2 (indolent and aggressive cancer cells found), and 3–5 (cancer cells found predominantly aggressive). For the purpose of developing a screening test (no cancer vs. any form of cancer), samples with any ISUP grade > 0 were classified as cancer. For the purpose of developing a surveillance test, samples with ISUP grades ≤ 2 were classified as indolent and samples with ISUP grade ≥ 3 were classified as aggressive.

### 2.3. Murine Models of Prostate Cancer

All of the procedures conducted were approved by the Indiana University Animal Care and Use Committee (protocol #SC311R) and complied with the Guiding Principles in the Care and Use of Animals, supported by the American Physiological Society (APS). Ten male C57BL/six mice (~8 weeks old) received transgenic adenocarcinoma mouse prostate (TRAMP-C2) cancer cells (approximately 0.1 million cells in 20 mL PBS) to the proximal tibia. Urine was collected and all of the tumor-bearing mice were sacrificed on day 21 after tumor injection. Prior to tumor injection, urine from the mice was obtained to serve as the healthy control group. The mice were caged at room temperature and fed the same diet (mouse chow ad libitum) during the experiment. The urine was collected over dry ice using Pasteur pipettes into glass centrifuge tubes. Furthermore, 75 μL aliquots of urine were transferred to a 10 mL headspace vial and stored in a −80 °C freezer before SPME GC-MS QTOF analysis was implemented.

### 2.4. Human and Mouse Urine Sample Processing

The samples from the patients were collected in polypropylene cups, sealed with parafilm, refrigerated, deidentified, and transported on ice to Indiana University–Purdue University Indianapolis (IUPUI) where the samples were aliquoted into headspace vials (2.5 mL urine) and stored at −80 °C until SPME GC-MS QTOF analysis. The GC-MS assays were randomized but stratified so that the samples with the same ISUP grade were not run consecutively. The samples were defrosted 30 min prior to SPME coupled to GC-MS and saturated with salt (0.9 g NaCl in 2.5 mL urine). Additionally, all of the urine was pH corrected to 6.5–7 with small amounts of 1M NaOH as saturating with salt lowers the pH of samples differentially, which undesirably varies the VOC profile sampled through SPME [50]. Regarding the analysis of urinary VOCs in the murine model of prostate cancer, the urine was defrosted 30 min prior to SPME GC-MS analysis and treated with GHCl in a 1:1 volumetric ratio. This is significant as GHCl denatures the major urinary proteins (MUPs) in mouse urine that bind VOCs in hydrophobic pockets [51].

### 2.5. SPME GC-MS QTOF Protocols

Prior to daily runs, the SPME fiber was preconditioned at 250 °C for 10 min. Vials with urine were incubated at 60 °C and agitated at 250 RPM for a total of 60 min, with extraction by SPME during the last 30 min. After extraction, the SPME fiber was injected into the inlet of the GC-MS QTOF which was held at 250 °C to thermally desorb the VOCs into the system. The oven temperature program utilized an initial temperature of 40 °C held for 2 min, followed by a ramp of 8 °C/min to 100 °C, 15 °C/min to 120 °C, 8 °C/min to 180 °C, 15 °C/min to 200 °C, and lastly 8 °C/min to 280 °C. The MS transfer line temperature was held at 250 °C during the chromatographic runs. Identical SPME GC-MS procedures (other than sample pretreatment) were implemented for the analysis of VOCs in mouse and human urine. External analytical reference standards in the form of high-density polyethylene (HDPE) were analyzed daily and demonstrated a high degree of instrumental and method reproducibility. Before analyzing mouse and human urine samples for prostate cancer biomarkers, UTAK urine standards were ran using a DVB/CAR/PDMS SPME fiber and a DVB/Carbon Wide Range/PDMS SPME arrow to determine which SPME type would be more effective in preconcentrating urinary VOCs.

### 2.6. Data Screening and Chemometric Analysis

Using a previously described method [48,49], chromatographic deconvolution and spectral alignment were implemented through MassHunter Profinder for the mouse and human urine samples independently. Alignment was verified by observing the retention time spread for each molecular feature, and compounds with high retention time variation were eliminated from the matrix. Spectrally aligned features that clearly contained two distinct molecular subpopulations were removed. VOCs present in fewer than 50% of samples in at least one class were also discarded. Features that were silica-based due to interaction with the SPME fiber and/or column were rejected, as were VOCs that were known exogenous pollutants and plasticizers. Preprocessing of the data matrices continued by normalizing each integrated VOC signal by relative abundance to account for variations in urine flow rate and concentration due to water intake. Two-tailed Student’s *t*-tests were implemented on the normalized data to screen for VOC differences between cancer and no cancer in both human and murine models. Univariate significance testing was also undertaken to identify VOCs differentially expressed in aggressive prostate cancer for human urine analysis.

Regarding multivariate analysis, subsets of VOCs were subject to principal component analysis (PCA) to observe global patterns in the data. PCA was implemented for the mouse and human urine samples independently. PCA reduces data dimensionality and identifies outliers, but this multivariate statistical technique is unsupervised and therefore may not differentiate sample classes well if the distinguishing features for the disease are not the ones with maximum variance in all samples [52]. In such cases, supervised methods such as linear discriminant analysis (LDA) are used to separate samples when reliable class labels are available. Supervised iterative LDA builds the model using a forward feature selection method and has been previously utilized for identifying VOC biosignatures of cancer [47,49,53]. Starting with the set of screened VOCs, LDA was performed on all permutations of three compounds, with it finding the three VOCs with the highest ability to distinguish two classes. Next, by reserving one of these three and re-analyzing, the best four compounds are found, and the process continues. The final set of compounds is obtained when the addition of more VOCs does not improve the cross-validated classification accuracy. The stability of the LDA models was tested by data perturbation [54]. To perturb the data set, a hold-out scheme was employed using the Matlab classifier application. In this system, a fraction (one fifth for five-fold cross-validation) of the samples was excluded to train the data set, and the held-out samples were used to test the model. The hold-out method was repeated using different randomly held out samples (1000 times) and an average receiver operating characteristic (ROC) curve was generated. Only the human urine sample data set was analyzed using supervised multivariate statistical methods.

## 3. Results

### 3.1. SPME Optimization

**Figure 1:**
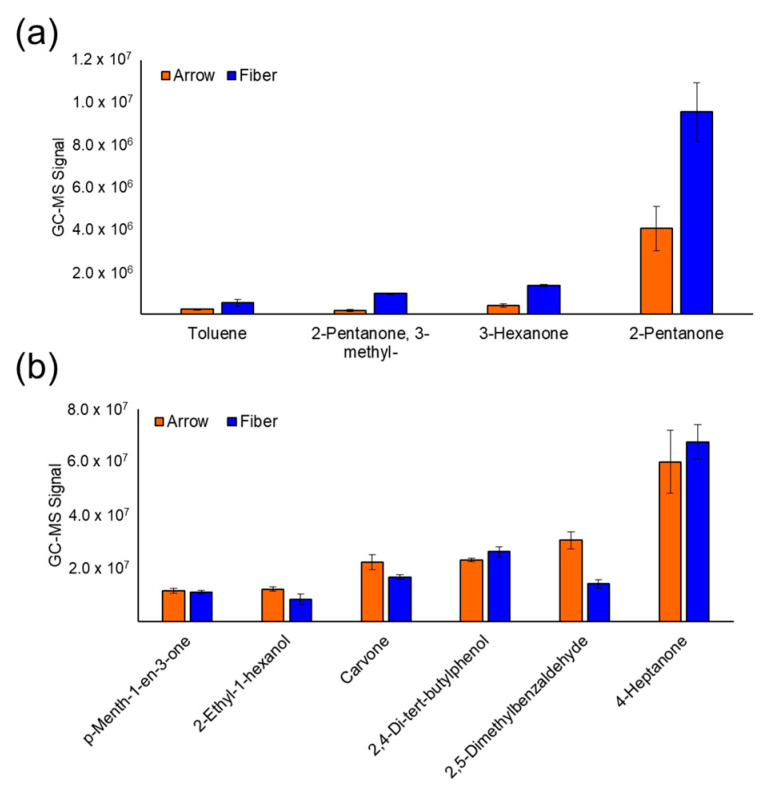
(**a**) Bar charts of GC-MS signals for VOCs with a relatively low molecular weight demonstrate that SPME fibers have higher extraction efficiency toward small analytes. (**b**) GC-MS signals of VOCs with a slightly higher molecular weight shows that the SPME arrow has slightly higher sensitivity for other analytes.

Prior to analyzing the samples, UTAK urine standards were analyzed using SPME fibers and arrows to identify the optimal extraction method. Here, DVB/CAR/PDMS SPME fibers were quantitatively compared to DVB/Carbon Wide Range/PDMS SPME arrows. Both SPME devices were analyzed using the same extraction protocol, and the results showed that after chromatogram deconvolution, there were no significant differences in the number of VOCs detected or the total integrated GC-MS signal (Supplementary Figure S1a,b). Upon integrating individual VOC signals, it was realized that the SPME fiber was much more sensitive toward VOCs with a relatively low molecular weight. For example, the fiber could successfully extract analytes including acetone, ethyl acetate, 2-butanone, and chloroform. These VOCs could not be detected when extracting the urine standards with the SPME arrows. The SPME fiber also extracted 2-pentanone, 3-hexanone, 3-methyl-2-pentanone, and toluene with higher sensitivity relative to the arrow (Figure 1a). Regarding VOCs with a higher molecular weight, varying results were identified in the optimization experiments. For example, the SPME arrow was slightly more sensitive to 2,5-dimethylbenzaldehyde, carvone, 2-ethyl-1-hexanol, and bornyl acetate. The SPME fiber on the other hand was slightly more sensitive to 2,4-di-tert-butylphenol and no significant differences were identified for 4-heptanone and p-menth-1-en-3-one (Figure 1b). Therefore, it was decided for the SPME fiber to be used for prostate cancer VOC biomarker discovery.

### 3.2. Patient Recruitment and Urine Collection

Human subjects (*n* = 162) meeting the inclusion and exclusion criteria provided consent and donated urine specimens prior to prostate cancer biopsies or prior to radical prostatectomy at the Richard L. Roudebush VAMC. Biopsy results from the subjects’ medical records were used to classify samples by ISUP grade and GS. The patient group is not random as all the men presented were scheduled for and underwent biopsies mainly due to elevated PSA levels. In total, 67 patients had negative biopsy results and 95 had positive prostate cancer biopsy results. Of those diagnosed with prostate cancer, there were 38 men diagnosed with ISUP grade 1, 30 with ISUP grade 2, 8 with ISUP grade 3, 5 with ISUP grade 4, and another 14 patients diagnosed with ISUP grade 5. The men were subsequently stratified into the following sample classes: (1) no cancer (those with negative biopsies, ISUP grade 0), (2) prostate cancer (positive prostate cancer biopsy with ISUP ≥ 1), (3) indolent prostate cancer (ISUP grades 1 and 2), and (4) aggressive prostate cancer (ISUP grades ranging from 3 to 5). Regarding the murine model of prostate cancer, 8 urine samples were collected prior to tumor injection to serve as healthy controls and 9 samples were collected after tumor injection.

### 3.3. Mouse Urine VOC Analysis

**Figure 2:**
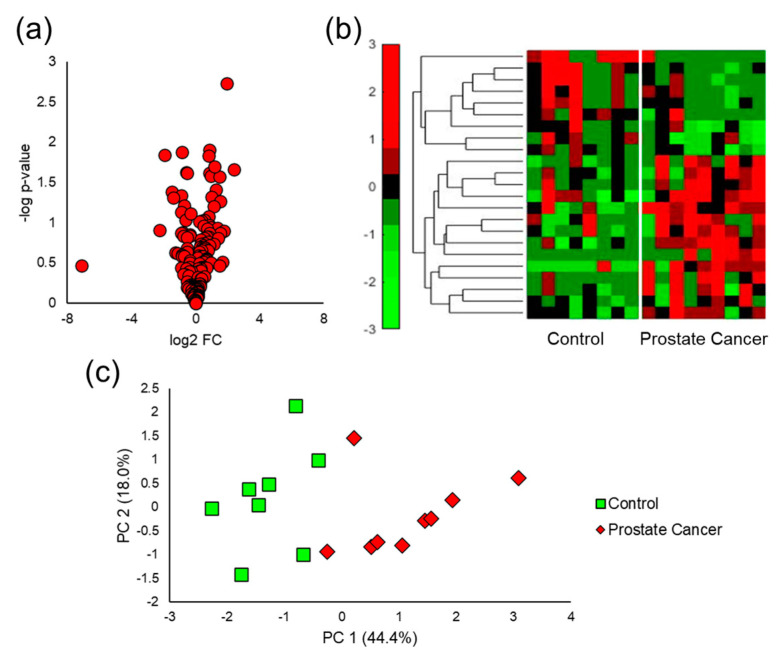
Univariate and multivariate chemometric analysis of mouse urine for prostate cancer VOC biomarker identification. (**a**) Volcano plot interpolating statistical significance as a function of log_2_ fold change shows more VOCs were upregulated by prostate cancer, (**b**) hierarchical heatmap of the VOCs differentially expressed shows that VOCs have higher interclass variation, and (**c**) PCA of the top five VOCs completely distinguishes healthy controls from tumor-bearing mice with 100% accuracy.

After SPME GC-MS analysis of the mouse urine samples, spectral alignment and data screening procedures yielded a total of 161 VOCs for chemometric analysis. Of these VOCs, 16 molecular features were identified by the Student’s *t*-test to have a *p*-value < 0.05 and 25 had a *p*-value < 0.10. A volcano plot interpolating statistical significance as a function of log_2_ fold change (FC) can be observed in Figure 2a and shows that there were slightly more VOCs upregulated by prostate cancer relative to those downregulated. This was observed in the data set containing all VOCs, and the analytes which were identified to be differentially expressed due to induced prostate cancer. To visualize the VOC signals in each of the samples collected, a hierarchical heatmap was generated for all of the VOCs identified with a *p*-value < 0.10 (Figure 2b). The heatmap also demonstrates there were more upregulated VOCs compared to downregulated features. In general, the VOCs in the heatmap display low intraclass variation and high interclass variation. Interestingly, the VOCs upregulated by prostate cancer in the mouse model had lower variations within the healthy control samples relative to the downregulated compounds. Lastly, unsupervised PCA was implemented on the five VOCs with the lowest *p*-values and the first two principal components accounted for 62.4% of the variation present in the sample data. Prostate cancer mouse urine samples were distinguished from healthy controls with 100% accuracy in the two-dimensional PCA plot (Figure 2c), showing the discriminatory power of urinary VOCs.

### 3.4. Distinguishing Prostate Cancer in Humans

**Figure 3:**
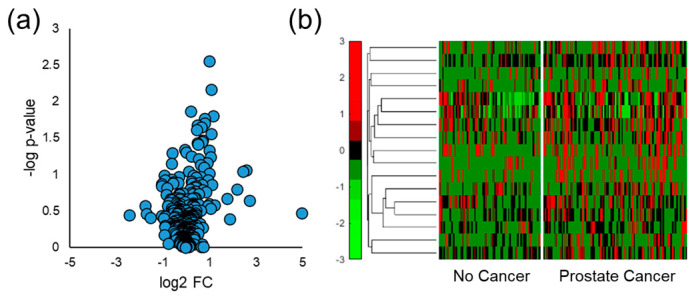
Univariate chemometric analysis of human urine for VOC biomarker identification regarding any grade/stage of prostate cancer. (**a**) The volcano plot shows that more VOCs are upregulated by prostate cancer in human urine relative to those that are downregulated, and (**b**) the hierarchical heatmap of VOCs identified with a *p*-value < 0.05 shows that the analytes have high variation regardless of prostate cancer diagnosis.

Human urine samples were also analyzed by SPME GC-MS, and similar data processing and analyses were implemented to distinguish men diagnosed with prostate cancer from those with negative biopsy results. Spectral alignment of qualified samples produced a matrix of 367 VOCs present in at least 50% of one of the four sample classes of interest (no cancer, prostate cancer, indolent prostate cancer, or aggressive prostate cancer). The Student’s *t*-test was used to probe differences in human urine due to the presence of any grade of prostate cancer and it identified 17 VOCs with a *p*-value < 0.05 and 29 features with a *p*-value < 0.10. Volcano plots were generated using these results and showed that most VOCs were upregulated in the urine samples collected from men with prostate cancer (Figure 3a). This especially holds true for VOCs identified with relatively low *p*-values and high absolute log_2_ FC values. Hierarchical heatmaps were made for VOCs that had a *p*-value < 0.05, and they show that VOCs had relatively high variation within both the no cancer and prostate cancer sample classes (Figure 3b).

To identify patterns in the data, unsupervised multivariate statistical analysis was implemented in the form of PCA. Two-dimensional PCA was tested with different permutations and combinations of VOCs, and limited separation of prostate cancer from the no cancer sample class was observed. For example, when PCA was implemented on features identified with a *p*-value < 0.05, the first two principal components accounted for 27.2% of the variation in the sample data and there was a large degree of overlap between the prostate cancer and no cancer samples (Supplementary Figure S2a). Even though PCA could not separate the sample classes with high accuracy, the first principal component showed higher statistical significance relative to any individual VOC (Supplementary Figure S2b). Regardless, PCA demonstrates poor sensitivity and specificity values, indicating that unsupervised multivariate analysis was not sufficient for accurate prostate cancer classification.

**Figure 4:**
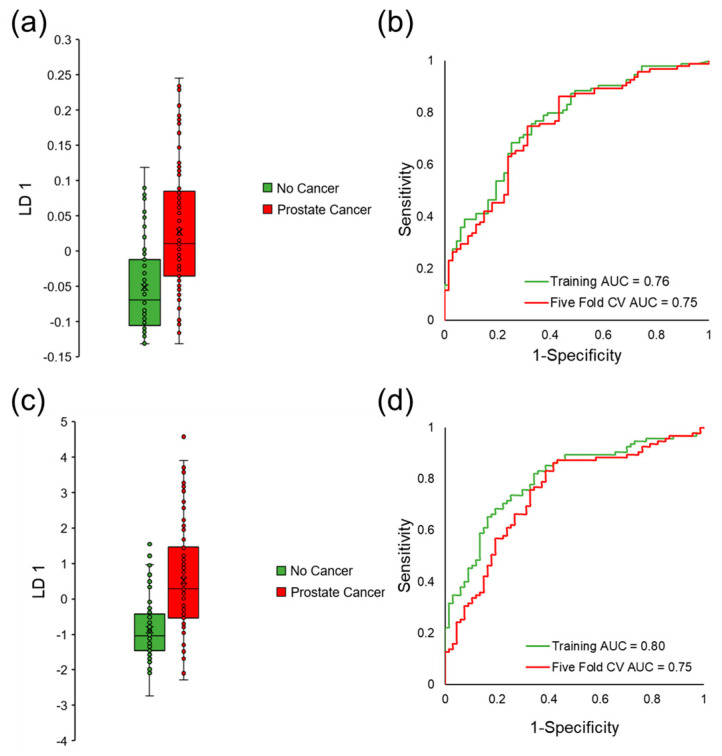
Supervised multivariate analysis of human urine to identify a biosignature of VOCs that could classify prostate cancer through LDA. (**a**) LD 1 scores for a model of six urinary volatiles that separates any grade of prostate cancer with (**b**) ROC AUC equal to 0.76 in the training data set and 0.75 in the cross-validated data set. (**c**) LD 1 scores for an alternative model of seven urinary VOCs that could distinguish prostate cancer with (**d**) ROC AUC equal to 0.80 in the training data and 0.75 in the cross-validated data set.

Therefore, forward feature selection coupled to LDA was implemented on qualified VOCs to build a predictive classification model and identify a biosignature of VOCs that classifies prostate cancer. This method identified six VOCs that discriminated between sample classes with an area under curve (AUC) equal to 0.76, sensitivity equal to 76%, and specificity equal to 67% in the training data set. The average five-fold cross-validation AUC was equal to 0.75 (sensitivity = 75% and specificity = 69%), which demonstrated that the model was not overfit. Linear discriminant 1 (LD 1) scores and ROC curves for this classification model can be observed in Figure 4a,b. Because the model was not overfit, the team continued with forward feature selection to add more VOCs to the model and increase the cross-validated classification accuracy. The algorithm selected a slightly different panel of seven VOCs that had higher classification accuracy in the training data (AUC = 0.80, sensitivity = 82% and specificity = 66%). Even though higher accuracy was observed in the training data, there were no increases in AUC values when the model was perturbed by five-fold cross-validation (AUC = 0.75, sensitivity = 82%, and specificity = 61%), indicating that this model is more overfit relative to the previously presented biosignature. LD 1 scores and ROC curves for this model can be observed in Figure 4c,d.

### 3.5. Stratifying Aggressive Prostate Cancer

**Figure 5:**
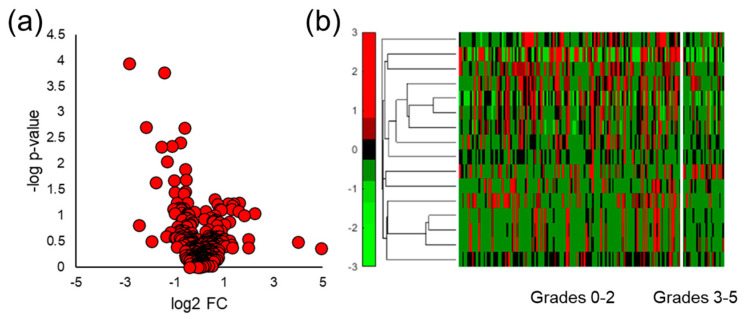
Univariate chemometric analysis of VOCs in human urine to distinguish aggressive grades of prostate cancer. (**a**) The volcano plot shows that more VOCs are downregulated by aggressive prostate cancer in human urine, and (**b**) the hierarchical heatmap of VOCs identified with a *p*-value < 0.05 shows that the analytes have high variation regardless of sample class.

VOCs in human urine headspace were also analyzed by similar chemometric approaches to distinguish aggressive prostate cancer from indolent prostate cancer and men with negative prostate cancer biopsies (no cancer). Here, differences in VOC expression were probed between patients with ISUP grades 0–2 and men with ISUP grades 3–5. Univariate analysis was implemented on the same data matrix of VOCs and identified 16 VOCs with a *p*-value < 0.05 and 40 volatiles with a *p*-value < 0.10. As opposed to the previous analysis distinguishing any grade of prostate cancer from the healthy controls, volcano plots for this statistical comparison demonstrated that there were more differentially expressed VOCs downregulated in aggressive prostate cancer samples relative to those that were upregulated (Figure 5a). Hierarchical heatmaps were also constructed for the features identified by the Student’s *t*-test to visualize their normalized signals in all of the samples (Figure 5b). Similar to the prostate cancer results, these VOCs also displayed high intraclass variation, indicating that these molecules alone may not have a high ability to stratify aggressive prostate cancer.

Next, multivariate analyses were implemented to assess the ability of multiple VOCs to separate aggressive prostate cancer. Unsupervised approaches through PCA were utilized in a similar fashion as previously shown for prostate cancer samples. The first two principal components explained 37.7% of the variation in the samples when PCA was used to reduce data dimensionality for the VOCs with a *p*-value < 0.05 (Supplementary Figure S3a). Even though the first two principal components accounted for more variation in the data relative to the PCA results for separating prostate cancer, the aggressive cancer samples were not accurately stratified from indolent cancer and no cancer. The first principal component alone accounted for 20.2% of the variance (Supplementary Figure S3b) in the data and aggressive cancer displayed significant differences when compared to the other samples (*p*-value = 0.0001). Unlike the PCA for prostate cancer, PCA of aggressive cancer did not display greater significant differences relative to individual VOCs alone, as the VOC with the lowest *p*-value was an aromatic compound and had a similar *p*-value when compared to the first principal component.

**Figure 6:**
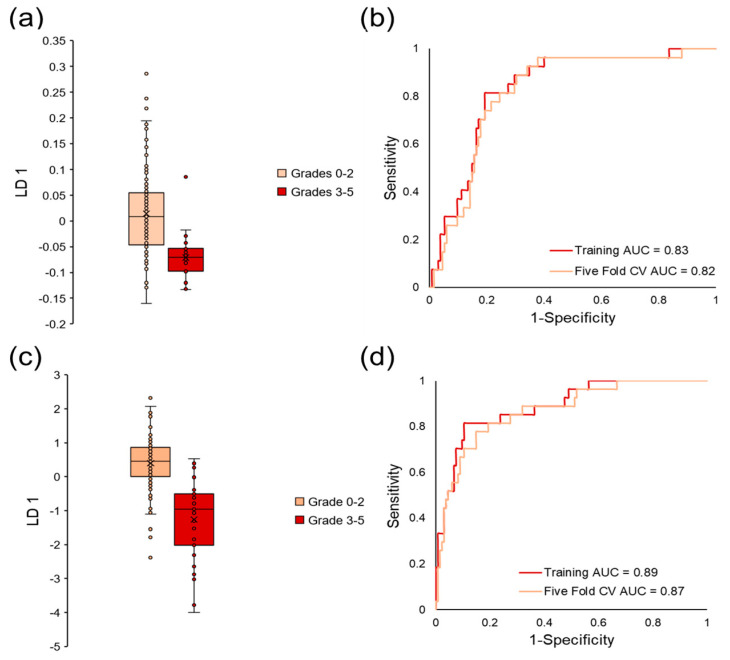
Supervised multivariate analysis of human urine to identify a biosignature of VOCs that could classify aggressive prostate cancer through LDA. (**a**) LD 1 scores for a model of six urinary volatiles that could classify aggressive tumors with (**b**) ROC AUC equal to 0.83 in the training data set and 0.82 in the cross-validated data set. (**c**) LD 1 scores for an alternative model of seven urinary VOCs that could stratify aggressive prostate cancer with (**d**) ROC AUC equal to 0.89 in the training data and 0.87 in the cross-validated data set.

Because PCA did not provide adequate stratification of aggressive prostate cancer, forward feature selection coupled to LDA was implemented on the screened data. This approach initially produced a predictive classification model of six VOCs that could distinguish prostate cancer with an AUC equal to 0.83 in the training data set (sensitivity and specificity = 81%). Five-fold cross-validation was performed 1,000x and the average AUC was equal to 0.82 (sensitivity = 81% and specificity = 76%) which demonstrated that the model was not overfit. Figure 6a,b show the LD 1 scores and ROC curves for this classification model. Analysis continued by identifying an alternative biosignature of seven VOCs that could distinguish aggressive prostate cancer with even higher accuracy. This new model demonstrated an AUC equal to 0.89 with relatively higher specificity values in the training data set (sensitivity = 81% and specificity = 89%). The model of VOCs also showed a high degree of stability, as the average five-fold cross-validated ROC curve displayed an AUC equal to 0.87 with a sensitivity equal to 78% and a specificity of 85% (LD 1 scores and ROC curves shown in Figure 6c,d).

### 3.6. Comparing VOC Biomarkers in Mouse and Human Urine

**Figure 7:**
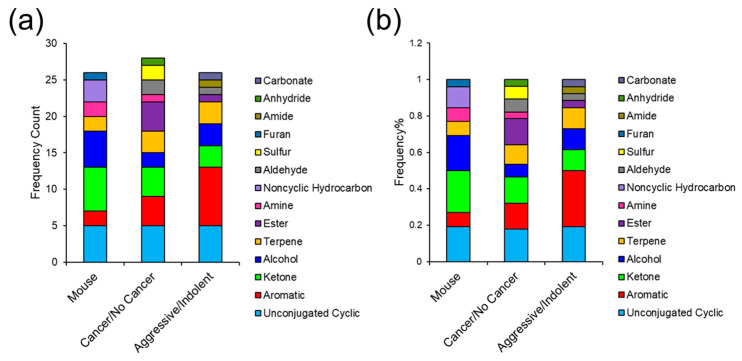
(**a**) Functional group frequency and (**b**) functional group frequency ratio of urinary VOCs identified to be differentially expressed (*p*-value < 0.05) by prostate cancer in the mouse model and in humans. The same is also displayed for urinary VOCs in humans that were dysregulated by aggressive prostate cancer.

Once urinary VOCs were analyzed for prostate cancer biomarkers in mice and humans independently, the results were compared. First, the specific functional groups were evaluated to assess the structural similarity of the candidate biomarkers in both models. VOCs with a *p*-value < 0.05 in the mouse model and human samples (including prostate cancer vs. no cancer and aggressive cancer vs. indolent/no cancer) were assessed for functional group frequency and functional group frequency ratio (Figure 7a,b). Overall, there was a high degree of similarities between the mouse and human urinalysis. The most common functional groups detected among both models consisted of nonaromatic (unconjugated) cyclics, aromatics, ketones, terpenes, and esters. Aromatic VOCs showed the highest abundance for aggressive prostate cancer comparison in humans, while ketones were the most prominent functional group detected in the mouse model. Nonaromatic cyclics were the most abundant VOC for prostate cancer stratification in humans but were also highly implicated in the other comparisons. Lastly, alcohols were more abundant in the mouse model and esters were more prominent in humans for the prostate cancer vs. no cancer univariate test.

## 4. Discussion

The VOCs emanating from urine in this study were assessed for their ability to stratify prostate cancer and aggressive prostate cancer, but there has been significant research on various cancer types and other medical conditions [55,56,57]. Some of the most well studied cancer types for VOC biomarker detection include prostate cancer, breast cancer, lung cancer, and bladder cancer. For example, some of the current authors have extensively published and identified VOC biomarkers in urine using mammary-tumor-induced murine models for breast cancer biomarker discovery. These studies show VOCs in urine (particularly aromatics, terpenes, and carbonyls) have the potential of determining tumor location [49], identifying the effect of antitumor therapies [48,58], and tracking mammary tumor progression over the course of time [47]. Breast cancer biomarkers have also been identified in human urine with varying diagnostic sensitivity/specificity values [50,59], and some of the most cited VOCs as potential biomarkers for breast cancer in these studies also consists of terpenes/terpenoids and ketones. More recently, Kure et al. built a classification model based on two urinary VOCs, 2-butanone and isopropanol (ketone and alcohol), which identified breast cancer with sensitivity and specificity equal to 93% and 83%, respectively [60].

The current analysis, however, focuses on prostate cancer detection through urinary VOCs and showed that method sensitivity is a significant factor to consider prior to biomarker discovery. The initial experiments in this study optimized SPME type by comparing fibers and arrows with similar chemical composition (DVB/CAR/PDMS fiber and DVB/Carbon Wide Range/PDMS arrow). The results revealed that even though SPME arrows have a larger surface area relative to fibers, there were no major differences in the total number of VOCs detected between the SPME types (Supplementary Figure S1), and arrows are not efficient for extracting low molecular weight VOCs (Figure 1). The rationale is that CAR has high adsorption affinity for low molecular weight VOCs and Carbon Wide Range does not (SPME Fiber Coating Selection Guide, provided by Sigma Aldrich). Even though the arrows did have higher sensitivity for a handful of larger VOCs, the SPME fiber was still able to preconcentrate these analytes with sufficient sensitivity. Therefore, for prostate cancer VOC biomarker discovery, it was decided for SPME fibers to be utilized.

After quantitative comparison of SPME fibers and arrows, mouse and human urine samples were analyzed for VOC biomarkers. The volcano plots showed that urinary VOCs were upregulated by prostate cancer in both mice and humans (Figure 2a and Figure 3a). When comparing aggressive cancers to the indolent and healthy controls, more VOCs were significantly downregulated relative to those that were upregulated (Figure 5a). There is no robust explanation as to why VOCs were more abundantly downregulated in aggressive cancer, but previous reports have shown that metabolites can be downregulated by cancer [47,48,49,61,62]. This may potentially be due to cancer cells using these VOCs to meet the increased energetic requirements for tumor growth [25]. Next, hierarchical heatmaps were constructed to visualize the expression of VOCs in the mouse and human urine data sets. VOCs identified in mouse urine (Figure 2b) had low intraclass variation and high interclass variation, but when analyzing human urine (Figure 3b and Figure 5b), VOCs had much higher variations within sample classes. This is expected as simplified models of disease including murine and in vitro studies offer experimental control of parameters that may undesirably vary urinary VOC profiles (diet, external environment, etc.). Nonetheless, simplified models of cancer do not take into account VOC differences that are due to the interactions between the tumor and the local microenvironment.

Multivariate analysis was implemented as a panel of VOCs may better reflect multiple pathways affected by a medical condition and can be cross-validated for accuracy better than a single compound [63]. Regarding mouse urine analysis, only unsupervised analysis was utilized due to the limited number of samples. Supervised multivariate analyses are more prone to overfitting when employed on small data sets [64]. PCA was capable of perfectly distinguishing prostate cancer in the mouse model (Figure 2c), but not in humans (Supplementary Figures S2 and S3). This is most likely because the highest amount of variation in the mouse data was due to prostate cancer, as there was a high degree of control over other variables. To identify a biosignature of VOCs to separate cancer/no cancer and aggressive/indolent prostate cancer in humans, iterative LDA was implemented. A panel of six VOCs was identified to distinguish prostate cancer with a cross-validated AUC equal to 0.75, and a separate set of seven VOCs had a cross-validated AUC of 0.75 (Figure 4). The panel of six VOCs was highly stable, while the panel of seven VOCs showed signs of overfitting. Iterative LDA also identified a panel of six VOCs that could distinguish aggressive cancer with a cross-validated AUC of 0.82 and a separate model of seven VOCs with a cross-validated AUC equal to 0.87 (Figure 6). Higher accuracies for classifying aggressive cancer were observed, which may be because there are metabolic similarities between indolent prostate cancer grades and healthy controls.

Mouse and human urine were initially analyzed independently, and then the results were compared. Functional group frequency analysis (Figure 7) indicated that mouse and human urine showed similar VOC functionality. The results from these analyses align with metabolic pathways dysregulated by prostate cancer. Carbonyls including volatile ketone bodies are produced when cytochrome P450 (CYP450) reduces hydroperoxides through fatty acid oxidation [65]. Increases in fatty acid oxidation are correlated with prostate cancer and have been studied as a dominant process that facilitates tumor growth by providing the necessary bioenergetics [66,67]. Moreover, P450 enzymes enable tumor progression as they activate carcinogens and reactive oxygen species [68,69]. Volatile terpenes/terpenoids have also been implicated as potential biomarkers and they are hypothesized to be biosynthesized via the mevalonate (MVA) pathway. Cancer requires increases in MVA products, including cholesterol and prenylated proteins [70], which have been used to develop inhibitors of farnesyl transferases, a common protein prenylator [71]. Increased MVA activity increases invasiveness in prostate cancer cells [70,72,73,74] and may play a role in cancer progression.

The VOCs implicated in this analysis also align with previous studies. For example, Khalid et al. identified carbonyls including pentanal, 3-octanone, and 2-octanone to be potential biomarkers of prostate cancer [25]. Other studies have also identified carbonyls as a rich source of potential biomarkers for prostate cancer [26,75]. Aromatic VOCs have also been identified to be biomarkers for prostate cancer. Interestingly, both our study and Lima et al. identified aromatics to be a potential source of urinary biomarkers for prostate cancer [26]. Benzaldehyde and other aromatics have also been reported in a number of human cancers [47,49,76]. The chemometric analysis in this study identified several terpenes/terpenoids in mice and men, and Lima et al. also identified a number of sesquiterpenes, bicyclic monoterpenes, and monoterpenoids as biomarkers for prostate cancer [26]. Additionally, Tyagi et al. identified dimethyl disulfide as a prostate cancer biomarker [75] and the present analysis implicated a structurally similar VOC. Lastly, Guest et al. identified seven VOCs which could classify prostate cancer patients with ISUP 5 from healthy controls [77]. Their panel consisted of many silica-based VOCs and a ketone (2-pentanone) along with other molecules that did not overlap with the VOCs identified in this study.

Limitations to the study include the fact that the subjects were not random but were selected for biopsy based on factors such as PSA levels, results from a digital rectal examination, and family history. Therefore, it is not possible to compute a positive predictive value or negative predictive value, as the subjects tested have different prevalence rates than the general population. Because many subjects had elevated PSA levels, it was not appropriate to compare the VOC results to the PSA test. Another limitation is that the VOCs found on these panels of biomarkers may not be exclusive to prostate cancer, as urine samples were not collected from patients with other types of medical conditions and because some of the VOCs identified as significant have also been found in other forms of cancer [65,78]. Different diseases including viruses and bacteriophages not only alter VOC expression [79,80,81] but can also increase the expression of genes correlating to tumor progression [82] which would require adding confounding variables (symptoms of viral infection) to the model for VOC biomarker interpretation. However, disease specificity is more of a concern for using a VOC test to identify cancer in the first instance but is not significant if a VOC test is being used for men already identified as having indolent prostate cancer but those concerned it may have progressed. While an abundant number of samples were collected from men with a negative biopsy (*n* = 67) and from those with indolent prostate cancer (*n* = 68), there were a relatively limited number of samples collected from those diagnosed with aggressive cancer (*n* = 27). Nevertheless, this study suggests urinary VOCs can discriminate prostate cancer with moderate accuracy and aggressive grades with even higher ability. Future studies can improve prostate cancer classification accuracy by controlling or exploring the effects of different confounding variables (diet, medications, and other lifestyle factors) and/or improving the sensitivity of current analytical instrumentation which would allow the detection of a denser VOC profile in urine headspace.

## 5. Conclusions

The results from this study show urinary VOCs can differentiate any form of prostate cancer with moderate accuracy and distinguish aggressive cancer with even higher sensitivity and specificity. The VOCs implicated as biomarkers for prostate cancer in the mouse and human urine samples were also shown to be structurally similar and are associated with expected metabolic pathways or have been previously identified as VOCs of inflammation or cancer. The use of a urine test to differentiate aggressive prostate cancer could reduce procedures on men identified with indolent prostate cancer, at a savings to the healthcare industry and the patients. With time and refinement, VOC assays may be coupled to other noninvasive assays to improve the clinical acceptance and predictive power of noninvasive tests. These panels of VOC biomarkers may one day be used to design a relatively low-cost, portable, smart gas sensor array capable of detecting urinary volatile biomarkers for prostate cancer diagnosis and monitoring cancer progression from indolent to aggressive. Rapid and point-of-care technologies for prostate cancer screening and distinguishing indolent/aggressive tumors may be able to improve medical decision-making, increase widespread testing, and decrease morbidity associated with prostate cancer in the future.

## Data Availability

The authors provide no restriction on the availability of the methods, protocols, instrumentation, and data utilized in the following article. Data will be available from the corresponding author upon reasonable requests.

## Supplementary Materials

The following supporting information can be downloaded at: https://www.mdpi.com/article/10.3390/cancers15041352/s1. Table S1. Comparison of accuracy, sensitivity, and specificity of different analytical methods that can be used to identify different molecular types of biomarkers for prostate cancer in biological samples. Figure S1. A comparison of SPME arrows and fibers shows no significant differences in performance regarding (a) the number of VOCs detected and (b) total integrated signal when utilized to extract a standard urine solution of VOCs. Figure S2. PCA of VOCs in human urine that were identified with a *p*-value < 0.05 when comparing men with and without prostate cancer. (a) The two-dimensional PCA plot explains 27.2% of the variation in the data but shows a high degree of overlap between the sample classes of interest. (b) One-dimensional PCA plot accounts for 15.3% of the variation in the data and shows greater statistical significance relative to any individual VOC. Figure S3. PCA of VOCs in human urine that were identified with a *p*-value < 0.05 when comparing men with aggressive prostate cancer to those with indolent grades and negative biopsy results. (a) The two-dimensional PCA plot explains 37.7% of the variation in the data but shows overlap and limited separation of aggressive prostate cancers from indolent and no cancer. (b) The one-dimensional PCA plot accounts for 20.2% of the variation in the data and shows statistical significance when stratifying aggressive prostate cancer.
