# Supplementary material for: Canine-Inspired Chemometric Analysis of Volatile Organic Compounds in Urine Headspace to Distinguish Prostate Cancer in Mice and Men
Source: Cancers (Basel). 2023 Feb 20;15(4):1352. doi: 10.3390/cancers15041352 (PMC9954105; doi:10.3390/cancers15041352)
Supplement: Supplementary file 1 [file cancers-15-01352-s001.zip › cancers-2150109-supplementary.pdf]

cancers

Supplementary Material

Canine-Inspired Chemometric Analysis of Volatile Organic Compounds in Urine Headspace to Distinguish Prostate Cancer in Mice and Men

Mark Woollam[1,2], Amanda P. Siegel[1,2], Adam Munshi[1], Shengzhi Liu[3], Sunil Tholpady[4], Thomas Gardner[4,5], Bai-Yan Li[3], Hiroki Yokota[6], and Mangilal Agarwal[1,2,7*]

[1] Integrated Nanosystems Development Institute, Indiana University-Purdue University, Indianapolis 46202, Indiana, USA
[2] Department of Chemistry and Chemical Biology, Indiana University-Purdue University, Indianapolis 46202, Indiana, USA
[3] Department of Pharmacology, School of Pharmacy, Harbin Medical University, Harbin 150081, China
[4] Richard L Roudebush Veterans Affairs Medical Center, Indianapolis 46202, Indiana, USA
[5] Department of Urology, Indiana University School of Medicine, Indianapolis 46202, Indiana, USA
[6] Department of Biomedical Engineering, Indiana University-Purdue University, Indianapolis 46202, Indiana, USA
[7] Department of Mechanical and Energy Engineering, Indiana University-Purdue University, Indianapolis 46202, Indiana, USA
* Correspondence: agarwal@iupui.edu

Abstract: Canines can identify prostate cancer with high accuracy by smelling volatile organic compounds (VOCs) in urine. Previous studies have identified VOC biomarkers for prostate cancer utilizing solid phase microextraction (SPME) gas chromatography–mass spectrometry (GC-MS) but have not assessed the ability of VOCs to distinguish aggressive cancers. Additionally, previous investigations have utilized murine models to identify biomarkers but have not determined if the results are translatable to humans. To address these challenges, urine was collected from mice with prostate cancer and men undergoing prostate cancer biopsy and VOCs were analyzed by SPME GC-MS. Prior to analysis, SPME fibers/arrows were compared, and the fibers had enhanced sensitivity toward VOCs with a low molecular weight. The analysis of mouse urine demonstrated that VOCs could distinguish tumor-bearing mice with 100% accuracy. Linear discriminant analysis of six VOCs in human urine distinguished prostate cancer with sensitivity = 75% and specificity = 69%. Another panel of seven VOCs could classify aggressive cancer with sensitivity = 78% and specificity = 85%. These results show that VOCs have moderate accuracy in detecting prostate cancer and a superior ability to stratify aggressive tumors. Furthermore, the overlap in the structure of VOCs identified in humans and mice shows the merit of murine models for identifying biomarker candidates.

Keywords: Prostate cancer biomarkers, volatile organic compounds (VOCs), gas chromatography (GC), mass spectrometry (MS), solid phase microextraction (SPME), chemometric analysis.

Table of Contents

Table S1. Comparison of accuracy, sensitivity and specificity of different analytical methods that can be used to identify different molecular types of biomarkers for prostate cancer in biological samples.

Figure S1. Comparison of SPME arrows and fibers shows no significant differences in performance regarding the (a) number of VOCs detected and (b) total integrated signal when utilized to extract a standard urine solution of VOCs.

Figure S2. PCA of VOCs in human urine that were identified with p-value < 0.05 when comparing men with and without prostate cancer. (a) Two-dimensional PCA plot explains 27.2% of the variation in the data but shows a high degree of overlap between the sample classes of interest. (b) One-dimensional PCA plot accounts for 15.3% of the variation in the data and shows greater statistical significance relative to any individual VOC.

Figure S3. PCA of VOCs in human urine that were identified with p-value < 0.05 when comparing men with aggressive prostate cancer to those with indolent grades and negative biopsy results. (a) Two-dimensional PCA plot explains 37.7% of the variation in the data but shows overlap and limited separation of aggressive prostate cancers from indolent and no cancer. (b) One-dimensional PCA plot accounts for 20.2% of the variation in the data and shows statistical significance when stratifying aggressive prostate cancer.

Table S1. Comparison of accuracy, sensitivity and specificity of different analytical methods that can be used to identify different molecular types of biomarkers for prostate cancer in biological samples.

Author with Citation #	Analytes of Interest	Biofluid	Method	Accuracy
Ashour *et al.* [28]	DNA	Tissue	NGS	Not Reported
Payne *et al.* [29]	DNA	Urine	PCR	Sensitivity = 78%, Specificity = 50%
Sunami *et al.*[30]	DNA	Blood	PCR	Sensitivity = 89%, Specificity = 91-100%
Zhou *et al.* [15]	Lipids	Blood	MS/MS	Sensitivity = 93.6%, Specificity = 90.1%
Buszewska-Forajta *et al.* [31]	Lipids	Tissue	MS/MS	Sensitivity = 82.2%, Specificity = 96.8%
Kyeong Min *et al.* [32]	Lipids	Urine	MS/MS	Not Reported
Skotland *et al.* [14]	Lipids	Urine	MS/MS	Sensitivity = 93%, Specificity = 100%
Li *et al.* [33]	Lipids	Urine	MS/MS	Not Reported
Sreekumar *et al.* [34]	Metabolites	Urine	GC-MS	Overall AUC = 0.71
Jentzmik *et al.* [35]	Metabolites	Urine	GC-MS	Overall AUC = 0.63
Zhang *et al.* [36]	Metabolites	Urine	LC-MS	Sensitivity = 83%, Specificity = 83.3%
Pinto *et al.* [37]	Metabolites	Urine	SF-MS	Overall Accuracy = 95%
Jones *et al.* [38]	Proteins	Blood	Immunoassay	Sensitivity = 58.9%, Specificity = 82.3%
Song *et al.* [39]	Proteins	Blood	Immunoassay	Sensitivity = 95.0%, Specificity = 78.2%
Al-Ruwaili *et al.* [40]	Proteins	Blood	MS/MS	Sensitivity = 73.3%, Specificity = 60%
Haj-Ahmad *et al.* [41]	Proteins	Urine	LC-MS	Sensitivity = 75%, Specificity = 50%
Daniel *et al.* [42]	RNA	Blood	PCR	Overall AUC = 0.95
Giglio *et al.* [43]	RNA	Blood	PCR	Overall AUC = 0.94
Roberts *et al.* [44]	RNA	Ejaculate	PCR	Overall AUC = 0.72
Van Neste *et al.* [45]	RNA	Urine	PCR	Sensitivity = 91%, Specificity = 36%
Taverna *et al.*[24]	Volatiles	Urine	Canine	Sensitivity = 98.6%, Specificity = 97.6%
Capelli *et al.* [46]	Volatiles	Urine	E-Nose	Sensitivity = 82%, Specificity = 87%
Khalid *et al.* [25]	Volatiles	Urine	GC-MS	Sensitivity = 74%, Specificity = 53%
Lima *et al.* [26]	Volatiles	Urine	GC-MS	Sensitivity = 78%, Specificity = 100%
Tyagi *et al.* [75]	Volatiles	Urine	GC-MS	Sensitivity = 76%, Specificity = 88%

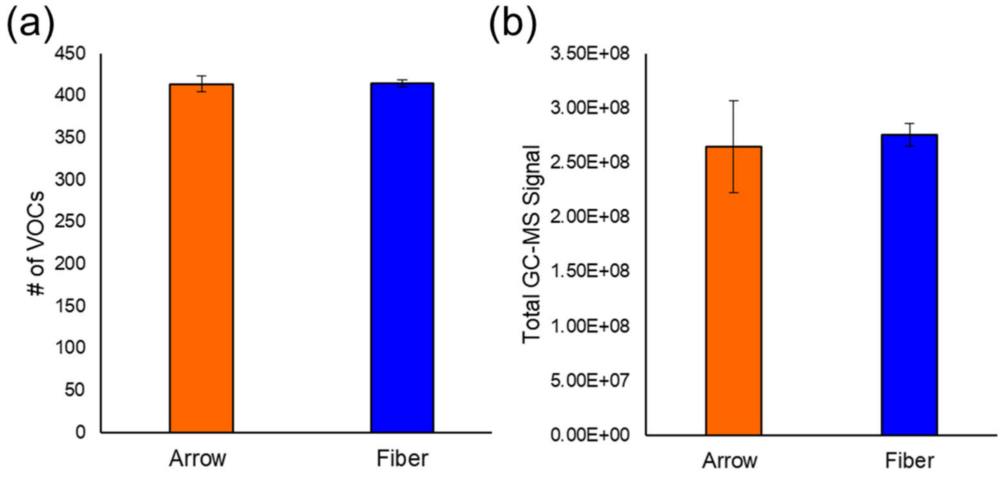

Figure S1. Comparison of SPME arrows and fibers shows no significant differences in performance regarding the (**a**) number of VOCs detected and (**b**) total integrated signal when utilized to extract a standard urine solution of VOCs.

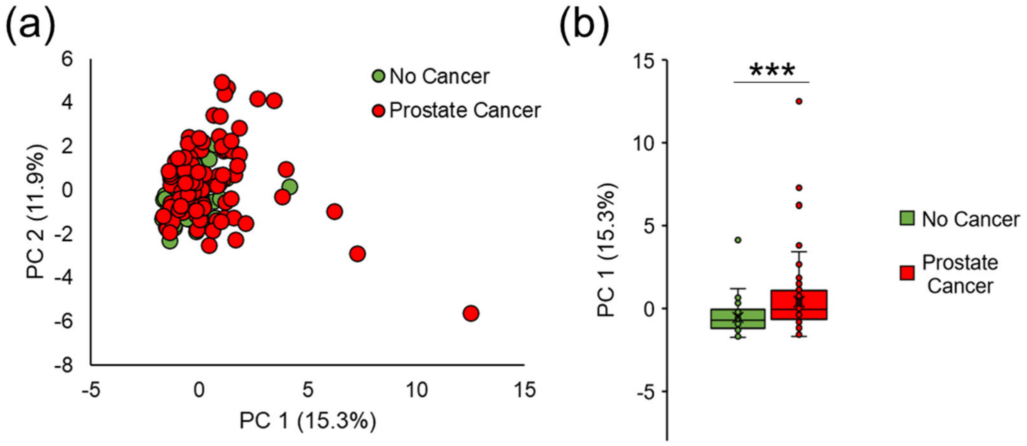

Figure S2. PCA of VOCs in human urine that were identified with p-value < 0.05 when comparing men with and without prostate cancer. (**a**) Two-dimensional PCA plot explains 27.2% of the variation in the data but shows a high degree of overlap between the sample classes of interest. (**b**) One-dimensional PCA plot accounts for 15.3% of the variation in the data and shows greater statistical significance relative to any individual VOC.

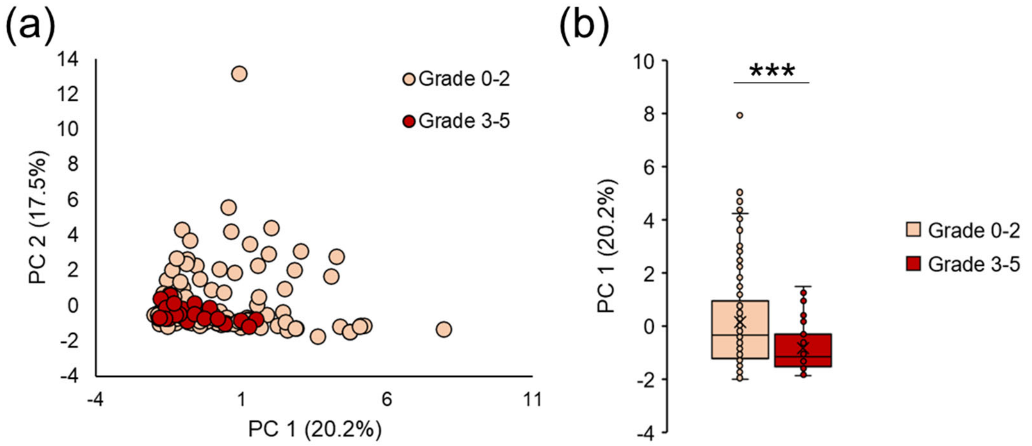

Figure S3. PCA of VOCs in human urine that were identified with p-value < 0.05 when comparing men with aggressive prostate cancer to those with indolent grades and negative biopsy results. (**a**) Two-dimensional PCA plot explains 37.7% of the variation in the data but shows overlap and limited separation of aggressive prostate cancers from indolent and no cancer. (**b**) One-dimensional PCA plot accounts for 20.2% of the variation in the data and shows statistical significance when stratifying aggressive prostate cancer.
